# Bone cement distribution may significantly affect the efficacy of percutaneous vertebroplasty in treating symptomatic Schmorl’s nodes

**DOI:** 10.1186/s12891-023-06575-8

**Published:** 2023-06-09

**Authors:** Kaiwen Cai, Guoqiang Jiang, Bin Lu, Kai Zhang, Kefeng Luo

**Affiliations:** 1 Department of Orthopaedic, The First Hospital Of Ningbo University, No. 247, Renmin Road, Jiangbei District, Ningbo, Zhejiang People’s Republic of China; 2 Institute of Orthopaedics, Ningbo University, No. 247, Renmin Road, Jiangbei District, Ningbo, Zhejiang People’s Republic of China

**Keywords:** Percutaneous vertebroplasty, Symptomatic Schmorl’s node, Low back pain, Clinical efficacy

## Abstract

**Purpose:**

Percutaneous vertebroplasty(PVP) has been widely used in treating symptomatic Schmorl’s nodes(SNs). However, there were still some patients with poor pain relief. At present, there is a lack of research to analyze the reasons for poor efficacy.

**Methods:**

Review the SNs patients treated with PVP in our hospital from November 2019 to June 2022, collect their baseline data. Reverse reconstruction software was used to calculate the filling rate of bone edema ring(R_f_). NRS score was used to evaluate pain and ODI to evaluate function. The patients were divided into remission group(RG) and non remission group(n-RG) according to symptom. In addition, according to the R_f_, they were divided into excellent, good and poor groups. Differences between groups were investigated.

**Results:**

A total of 26 vertebrae were included in 24 patients. When grouped according to symptoms, patients in n-RG were older, and surgical segments were tend to locate in lower lumbar spine. The proportion of Poor distribution was significantly higher. When grouped according to the cement distribution, the preoperative NRS and ODI of the three groups were comparable, but the NRS and ODI of Poor group were significantly worse than the Excellent and Good groups postoperatively and at the last follow-up.

**Conclusions:**

The cement distribution may significantly affect the efficacy of PVP in treating symptomatic SNs. We suggest that the bone edema ring should be filled as fully as possible to ensure the efficacy. In addition, advanced age and low lumbar lesions are also adverse factors for clinical outcomes.

## Introduction

Schmorl’s nodes(SNs) are not rare. As early as 1858, German anatomist Von Luschka first described this lesion [1]. To date, SNs have been frequently diagnosed clinically. The etiology of SNs is still controversial, possible causes include trauma, degeneration, vascular factors, Scheuermann’s disease, autoimmune diseases, etc. [2]. Nevertheless, the association between partial SNs and LBP has been revealed. In 1995, Takahashi et al. [3] found that bone marrow around to SNs showed low signal on T1-WI and high signal on T2-WI in MRI of all symptomatic SNs patients. Histological examination also confirmed the presence of significant bone marrow edema and inflammation in these areas. But in asymptomatic cases, the above imaging and histological changes were not seen. Subsequent similar studies have repeatedly confirmed this finding, that is, SNs with type I Modic changes are highly correlated with LBP [4, 5]. Local pathological changes, such as endplate cartilage rupture and subchondral bone edema, promote sustained pressure stimulation and release of inflammatory mediators, thereby reducing the efficacy of conservative treatment in these patients [5]. There is insufficient evidence to support the long-term efficacy of conventional therapies such as steroid injections, anti-TNF**α** antibodies, bisphosphonates, and radiofrequency ablation [7, 8].

Alternatively, some scholars have attempted to utilize percutaneous vertebroplasty (PVP) or percutaneous kyphoplasty (PKP) as treatment options for symptomatic SNs, with all reporting favorable long-term outcomes [9–14]. However, despite the positive results reported in both previous studies and our own clinical practice, a subset of patients still experience persistent pain following PVP. These cases present a challenging obstacle for further treatment and result in disappointing clinical outcomes. Regrettably, prior researches has not yet addressed this issue. This prompted us to conduct a retrospective study of patients who underwent PVP for SNs to explore the factors associated with poor outcomes. We hypothesized that the patient’s clinical out-come was correlated with the patient ‘s baseline characteristics or surgical intervention.

## Materials and methods

The ethical approval of the study (KY20191103) was issued by the ethics committee of The First Hospital Of Ningbo University. All methods were carried out in accordance with relevant guidelines and regulations. Informed consent signing is exempt because of the study’s retrospective study design.

### Patient selection

From November 2019 to June 2022, a total of 43 consecutive patients with type 1 Modic changes and SNs were diagnosed in our hospital and received PVP surgery. The medical records of them were reviewed. The inclusion criteria were as follows: (1) MRI confirms SNs with type 1 Modic changes, i.e. edematous rim around the nodes. (2) Localization of symptoms and signs of LBP consistent with imaging. (3) Patients with no clear history of trauma or only a history of mild exertional activity before onset. The exclusion criteria were as follows: (1) Confirmed fresh vertebral fracture, characterized by the presence of newly formed fracture lines, cortical defects or folds on preoperative computer tomography(CT) images. (2) Patients with spinal infections, tumors and congenital malformations. (3) Lost or dead during follow-up. (4) Spinal fractures or other spinal diseases that may affect outcomes occurred during the follow-up period. The inclusion and exclusion procedures were independently conducted by two experienced spinal surgeons, with any controversial cases being subjected to discussion in order to determine their eligibility.

After inclusion, baseline data were collected, including gender, age, mean lumbar bone mineral density T value ( BMD-T ), body mass index ( BMI ), surgical segment, bone cement volume, and follow-up duration.

### Surgical management

In general, patients took a prone position, after the C-arm fluoroscopy determined the target segment, local anesthesia was given. Then, pedicle puncture was performed, it should be noted that, unlike conventional PVP surgery, the puncture rods should point in the direction of the SNs position, we need to ensure that the perspective visible puncture needle (Kinetic, Shanghai, CHN ) tip near the edge of the SNs [6]. Then, polymethyl methacrylate (PMMA) bone cement (Tecres S.P.A., ITA) was prepared and the injection must completed within the low-viscosity wet-sand phase to low-viscosity wire-drawing phase. Confirmed the location of bone cement using sequential C-arm fluoroscopy to ensure that the nodes contour was covered with bone cement and that no intraspinal leakage occurred. In the event of intervertebral or paravertebral leakage, the injection process should be suspended, until the solidified strength of the leaked cement was sufficient to permit continued injection. All patients were required to remain supine in bed and monitored for four hours after surgery.

### Radiographic measurement

**Fig. 1:**
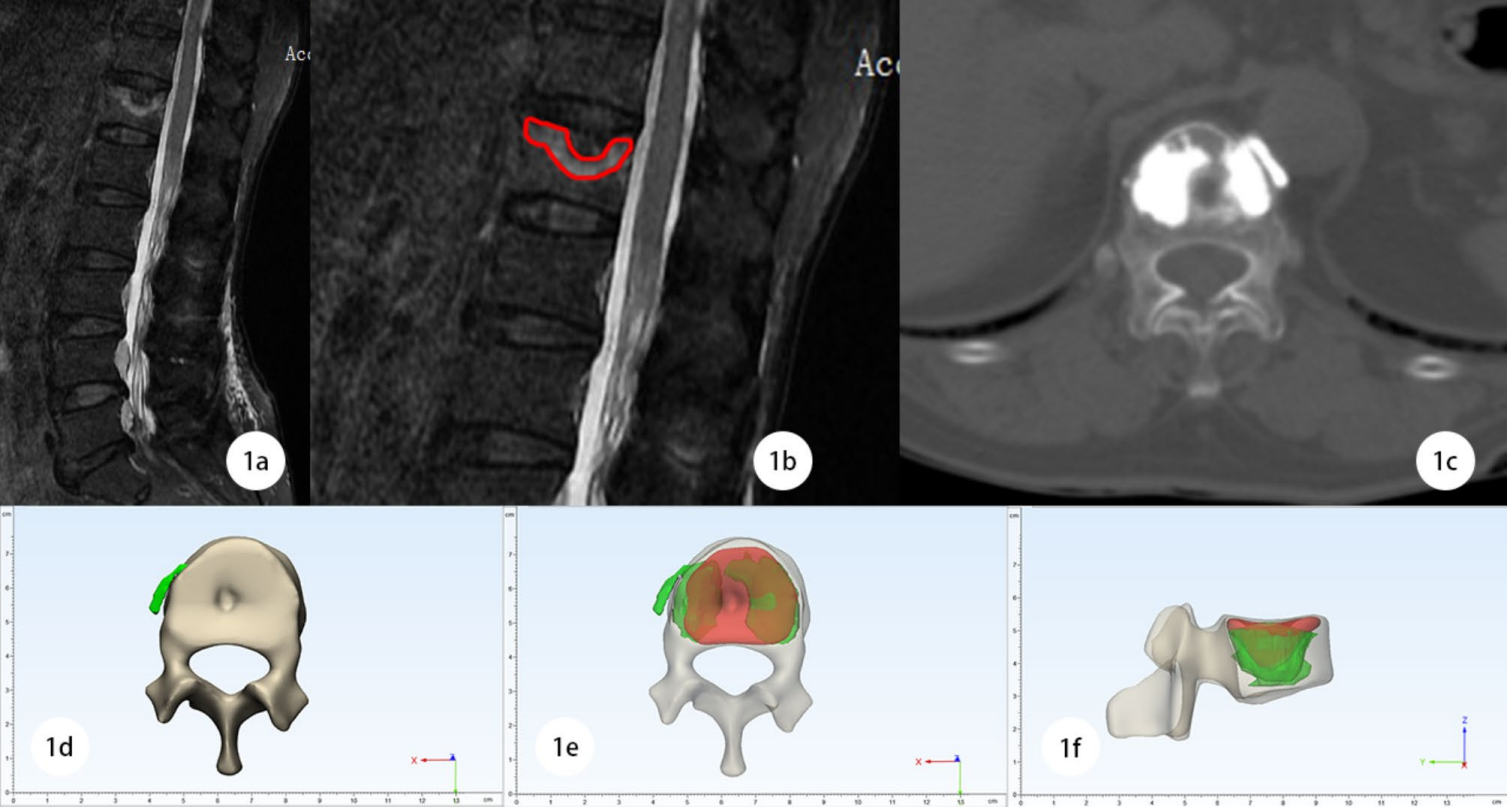
An enrolled case to illustrate the modeling method: 1a MRI fat suppression image of lumbar spine in a enrolled case showed SNs with Modic I changes in the cephalic endplate of T12 vertebra. 1b The boundary of bone edema ring is delineated (red circle). 1c Postoperative CT provides details of cement distribution. 1d Top view of reverse reconstruction model. 1e Reverse reconstruction of model top view (perspective), showing vertebra (yellow), bone cement (green), bone edema zone (red). 1f Reverse reconstruction model profile view (perspective), showing vertebra (yellow), bone cement (green), bone edema zone (red)

We used Mimics Research 21.0 and 3-matic Research 13.0 (Materialise, BEL) software to reconstruct the models of surgical segments for each patient, the range of the bone edema area was framed according to the range of high signal of the MRI fat suppression image (Fig. 1a,b). Then, the 3D model of the bone cement was reconstructed according to the postoperative CT (Fig. 1c). We matched both reconstruction models and calculated the proportion of bone edema area filled with bone cement (Fig. 1d,e,f). We used software to measure the volume of bone cement (V_cement_) and bone edema rings (V_edema_), performing boolean operations to obtain the volume of the bone edema ring filled with bone cement (${V_{(edema \cap cement)}}$), then we got the filling rate of the bone edema ring (R_f_). The calculation formula is as follows :

$${R_f} = \frac{{{V_{\left( {edema\ \cap\ cement} \right)}}}}{{{V_{edema}}}} \times 100\%$$

The grading criteria were as follows: (1) Excellent: R_f_ >70%; (2) Good: R_f_ = 40–70%; (3) Poor: R_f_ <40%. Furthermore, bone cement leakage or other abnormal distribution will be documented.

### Clinical outcomes data collection and grouping

Pain assessment using numerical rating scale(NRS) [15], collected at preoperative, postoperative and last follow-up period. Functional evaluation was performed using the oswestry disability index (ODI) [16], which was collected preoperatively and at last follow-up.

We divided patients into remission group(RG) and non-remission group(n-RG) according to their chief complaints at the last follow-up. The quantitative criteria for grouping were: RG group NRS ≤ 3, n-RG group NRS ≥ 4. For these two groups, we compared differences in their baseline characteristics, cement distribution and clinical outcomes. If differences in clinical outcomes were significantly associated with the distribution pattern of bone cement, we will further group according to the distribution pattern of cement, including: Excellent group, Good group and Poor group. To analyze the effect of bone cement distribution on clinical outcomes.

### Statistical methods

Statistical analysis was performed using SPSS 26.0 software ( IBM, NY, USA ). For measurement data, we use 2-independent samples T test. One-Way ANOVA was used to compare the differences between three groups, and LSD-T test was used for internal comparison. For enumeration data, we use Fisher exact probability method. For ranked data, the Mann-Whitney U test was used. P<0.05 was considered statistically significant.

## Results

### Patient inclusion results

A total of 43 consecutive patients who met the diagnosis were reviewed. During the follow-up period, 2 patients expired due to unrelated illnesses, 6 patients were lost to follow-up, and 11 patients experienced new osteoporotic vertebral compression fractures and were subsequently excluded from the study. Another 24 cases were finally included, with a total of 26 surgical vertebrae. The baseline characteristics of the patients were : age ( 70.5 ± 9.3 ) years, gender (M 6 / F 18), BMD-T ( -2.93 ± 0.87 ), BMI ( 22.5 ± 3.8 ) kg / m^2^, follow-up length from 8 to 38 months, with an average of ( 21.7 ± 10.3 ) months.

### Surgical findings and imaging grading

**Fig. 2:**
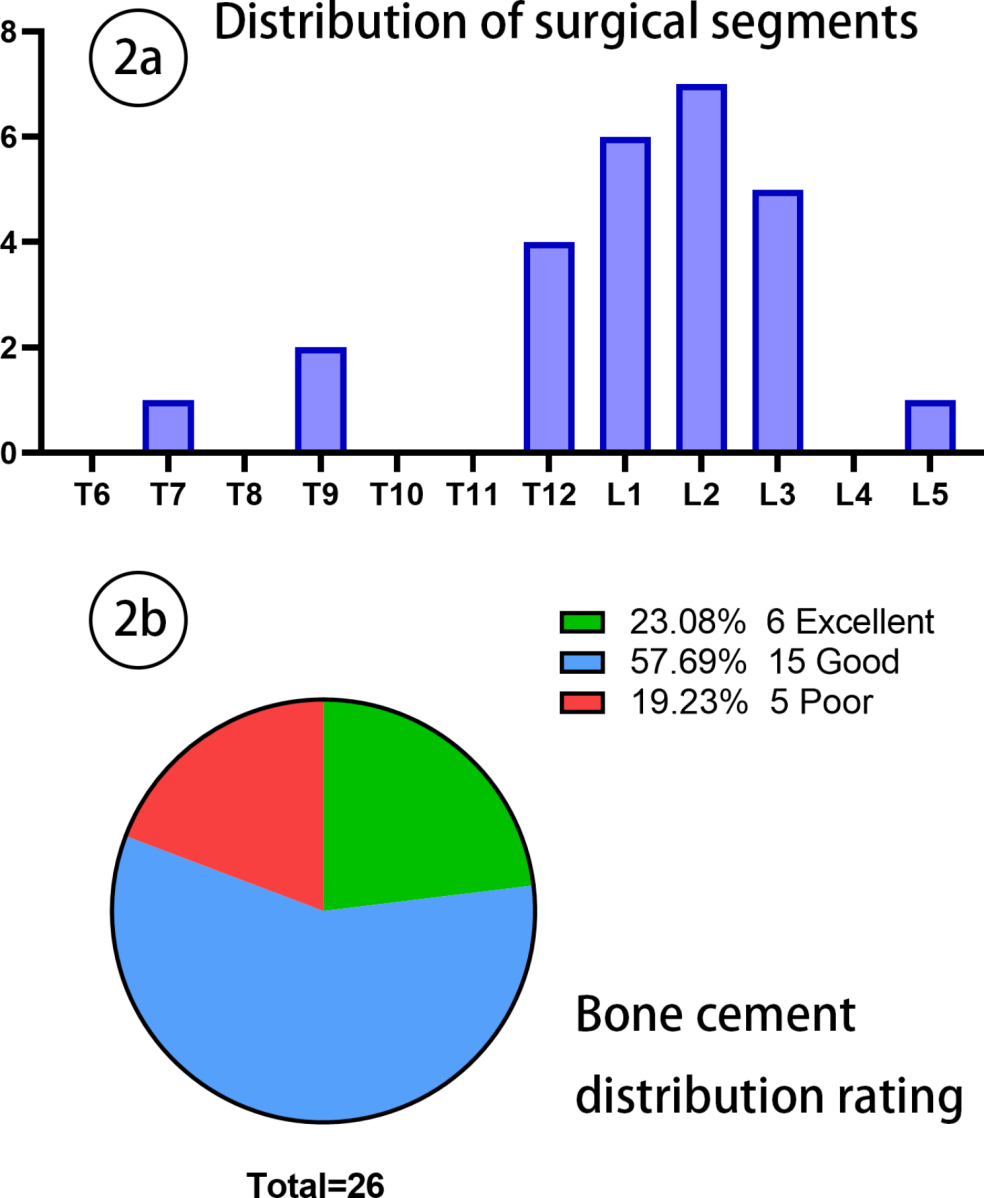
2a Frequency of surgical segment distribution. 2b Rating results of bone cement distribution

Of the 26 vertebrae, 3 were located in the thoracic segment ( T9 and above ), 17 in the thoracolumbar segment ( T10 to L2 ), and 6 in the lower lumbar segment ( L3 and below ). The segmental distribution details are shown in Fig. 2a. According to the R_f_ value, the bone cement distribution rating was : Excellent 6, Good 15, and Poor 5(Fig. 2b). We observed 5 cases of intervertebral disc leakage and 4 cases of paravertebral vascular leakage. There was no serious complications such as intraspinal leakage or pulmonary embolism been reported.

### Clinical efficacy

**Fig. 3:**
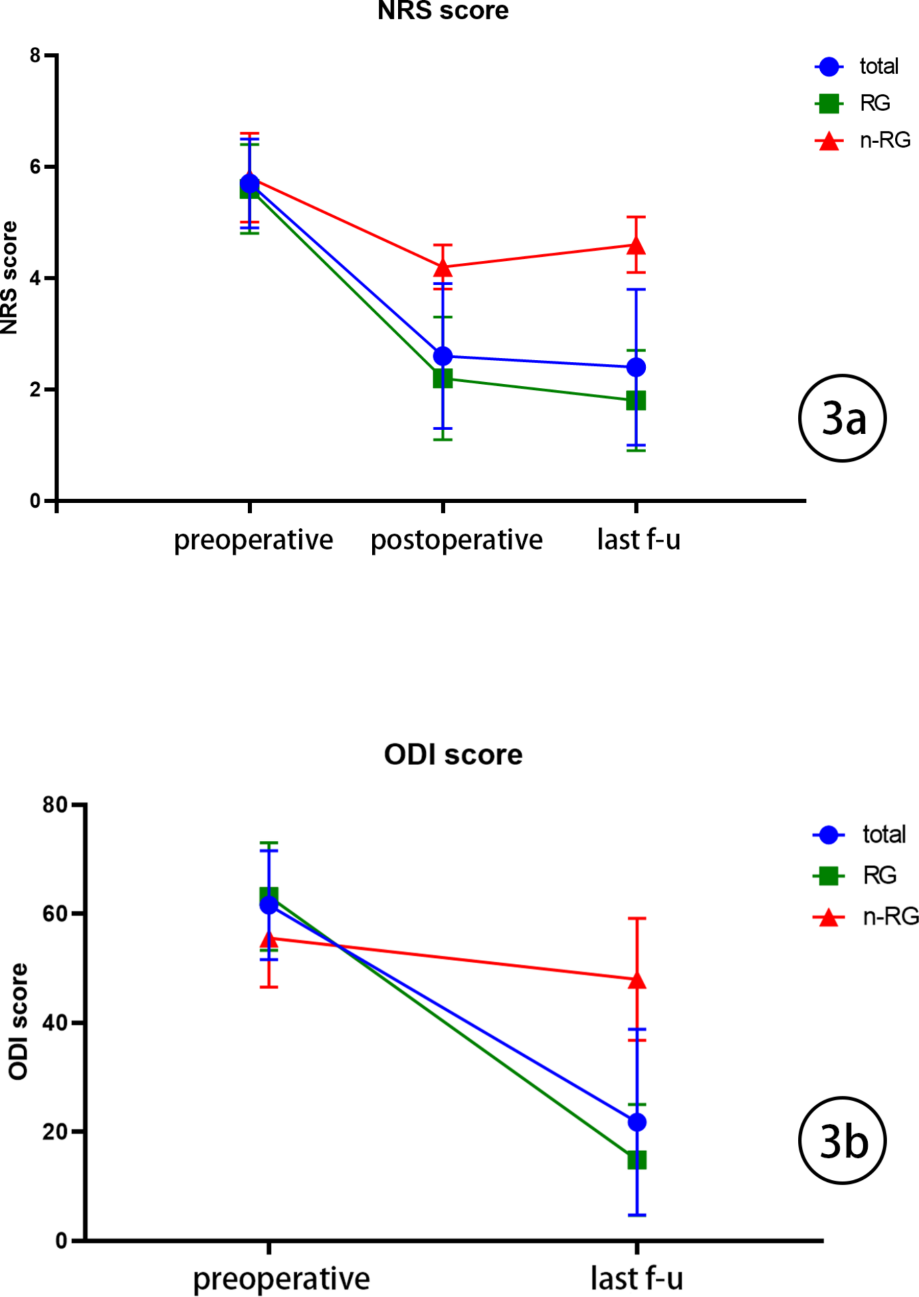
3a Line chart: NRS trend of all cases, RG and n-RG. 3b Line chart: ODI trend of all cases, RG and n-RG.

By the time of the last follow-up, a total of 19 patients reported significant alleviation of their LBP (NRS ≤ 3). Another 5 patients reported inadequate pain relief after surgery. (NRS ≥ 4), which continued to significantly limit their daily activities. Overall, NRS scores decreased from (5.7 ± 0.8) preoperatively to (2.6 ± 1.3) postoperatively and further improved to (2.4 ± 1.4) at the last follow-up visit. The ODI score also showed marked improvement, decreasing from (61.57 ± 10.01) preoperatively to (21.75 ± 17.08) at the latest follow-up assessment.(Fig. 3a,b).

**Table 1:** Comparison of baseline characteristics and clinical outcomes between RG and n-RG

	RG (n = 19)	n-RG (n = 5)	Statistical value	P value
Age (y)	68.2 ± 8.3	79.4 ± 7.8	t=-2.708	**0.013**
Gender(M/F)	3/16	3/2	/	0.078
BMD-T	-2.95 ± 0.91	-2.98 ± 0.75	t=-0.196	0.846
BMI(kg/m^2^)	22.35 ± 3.85	23.18 ± 4.15	t=-0.421	0.678
F-u duration (m)	20.7 ± 9.9	25.4 ± 12.2	t=-0.894	0.381
Segmental location (T/TL/L)	2/16/3	1/1/3	/	**0.037**
Cement volume (mm^3^)	6.34 ± 1.87	4.90 ± 1.24	t = 1.617	0.120
Cement distribution rating (Excellent/Good /Poor)	6/14/1	0/1/4	Z=-3.111	**0.002**
Intervertebral cement leakage (Y/N)	3/16	2/3	/	**0.270**
Preoperative NRS	5.6 ± 0.8	5.8 ± 0.8	t=-0.403	0.691
Postoperative NRS	2.2 ± 1.1	4.2 ± 0.4	t=-3.962	**0.001**
Last f-u NRS	1.8 ± 0.9	4.6 ± 0.5	t=-6.922	**<0.001**
Preoperative ODI	63.15 ± 9.87	55.56 ± 9.04	t = 1.554	0.134
Last f-u ODI	14.84 ± 10.18	47.98 ± 11.20	t=-6.357	**<0.001**

In comparing RG and n-RG, no significant differences were found in terms of gender, BMD-T, BMI, follow-up duration, bone cement volume, intervertebral leakage, preoperative NRS and preoperative ODI between the two groups. However, n-RG patients were significantly older than RG patients and had a higher incidence of lower lumbar spine involvement. Most notably, up to 80% of bone cement distribution was rated as “Poor” in n-RG compared to only 4.8% in RG (Table 1).

**Table 2:** Comparison of baseline characteristics and clinical outcomes between Excellent, Good and Poor groups

	Excellent (n = 6)	Good (n = 13)	Poor (n = 5)	Statistical value	P value
Age (y)	64.7 ± 11.0	71.1 ± 7.7	76.2 ± 8.7	F = 2.417	0.114
Gender(M/F)	1/5	3/10	2/3	/	0.681
BMD-T	-2.98 ± 1.24	-2.91 ± 0.79	-2.92 ± 0.73	F = 0.015	0.986
BMI(kg/m^2^)	18.7 ± 2.8	23.8 ± 2.9^*^	23.9 ± 4.4^*^	F = 5.545	**0.012**
F-u duration (m)	14.3 ± 5.7	22.9 ± 10.4	27.4 ± 11.0	F = 2.733	0.088
Segmental location (T/TL/L)	1/3/2	1/10/2	1/2/2	/	0.519
Cement volume (mm^3^)	6.14 ± 2.04	6.50 ± 1.78	4.70 ± 1.30	F = 1.892	0.176
Intervertebral cement leakage (Y/N)	1/5	3/10	1/4	/	1.000
Preoperative NRS	5.5 ± 1.0	5.6 ± 0.8	6.0 ± 0.7	F = 0.545	0.588
Postoperative NRS	2.5 ± 1.0	2.0 ± 0.9	4.4 ± 0.5^*,†^	F = 13.156	**<0.001**
Last f-u NRS	1.7 ± 1.0	2.0 ± 1.2	4.2 ± 0.8^*,†^	F = 9.351	**0.001**
Preoperative ODI	56.28 ± 10.30	61.54 ± 10.36	68.00 ± 5.38	F = 2.034	0.156
Last f-u ODI	12.57 ± 8.96	17.08 ± 13.05	44.88 ± 14.18^*,†^	F = 11.194	**<0.001**

^*^ Compared with ‘Excellent’ group, P < 0.05

^†^ Compared with ‘Good’ group, P < 0.05

Further, we grouped all patients according to the cement distribution rating. Since there were 2 patients with double surgical segments, and were all rated as ‘Good’, so we counted the two segments of each of them as one person to avoid repeated counting. We noted that the BMI of the Excellent group was lower in baseline data, while there was no significant difference in other baseline characteristics among the three groups. There was also no significant difference in preoperative NRS and ODI among the three groups. However, pain relief and functional recovery in Poor group were significantly worse than those in the other two groups after surgery and at the last follow-up, while the difference between the Excellent and Good groups was not significant. The intervertebral cement leakage rates were also comparable among the three groups (Table 2).

## Typical case presentation

### Case 1 (Group n-RG / poor)

**Fig. 4:**
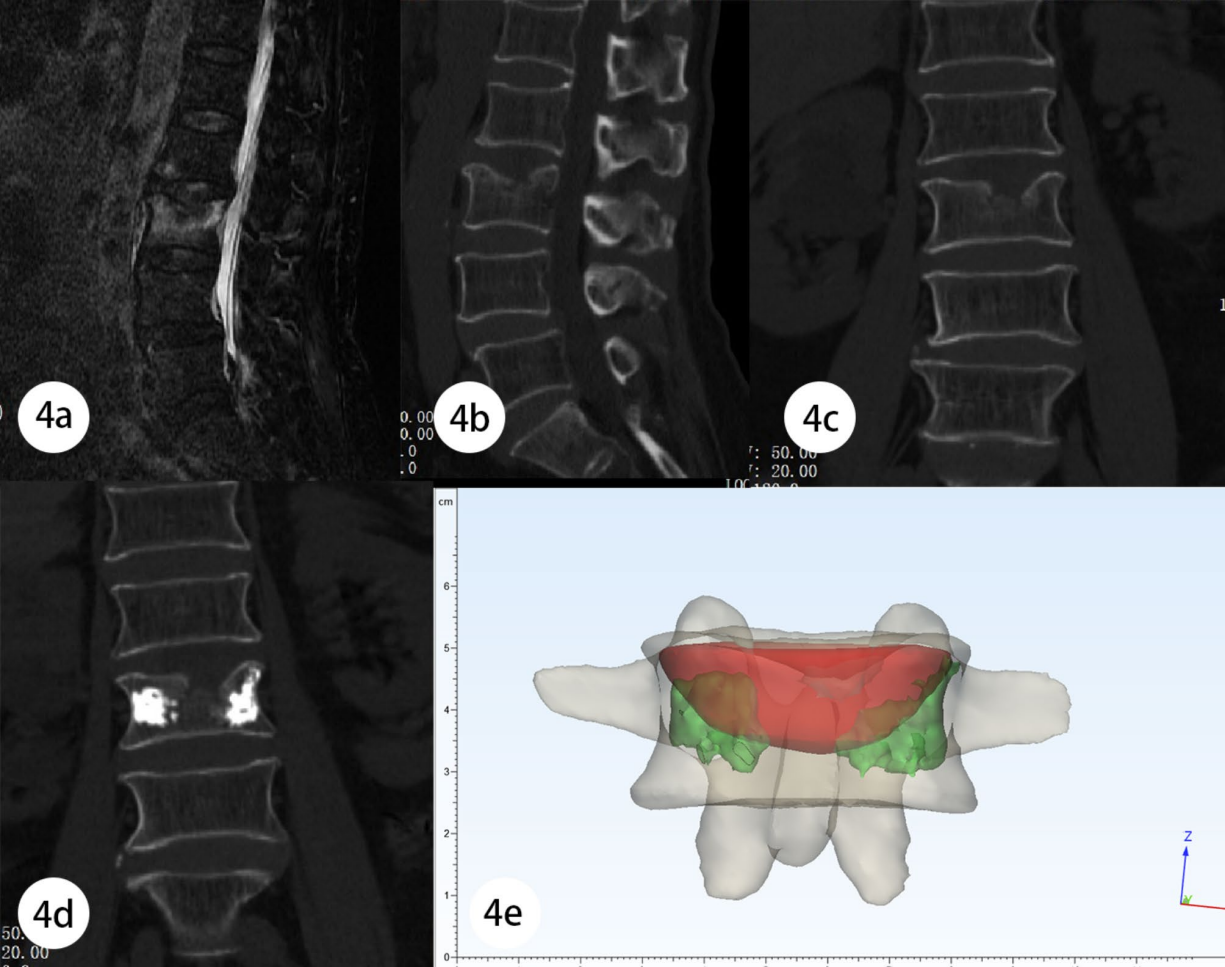
Case1: 4a The preoperative fat suppression image of lumbar spine MRI showed SNs with Modic I changes in the cephalic endplate of L3 vertebra. 4b、c SNs were displayed on sagittal and coronal images of preoperative CT. 4d Postoperative CT showed poor distribution of bone cement. 4e Reverse reconstruction model, calculated R_f_=13.85%

An 84 - year - old man complained of LBP for two weeks. This patient denied a recent history of trauma, but he had once carried a heavy weight of 10KG half a month ago and thereafter developed persistent LBP, which gradually worsened until, before admission, his NRS score of 6 and ODI of 66.67. Physical examination showed that the percussion pain of spinous process at L3 level was obvious, and there was no radiation pain in both lower limbs, skin sensation and muscle strength of both lower limbs was symmetrical and normal. The results of bilateral straight-leg raising test were negative. Preoperative MRI and CT confirmed a Schmorl ‘s node in the upper endplate of his L3 vertebra and a well defined ring of bone edema (Fig. 4a,b,c).

He underwent PVP surgery in our hospital, but this patient complained that his LBP improved very little after surgery. Referring to the coronal images of postoperative CT, we found that the distribution of bone cement was far from the boundary of SNs, and the amount of bone cement injection was insufficient (Fig. 4d). With the help of the reconstruction model, we calculated his R_f_ = 13.85% (Fig. 4e). Therefore, the patient was classified into the ‘Poor’ group. Until the last follow-up at nine months after surgery, this patient still had obvious LBP ( NRS = 4 ) and limited activities of daily living ( ODI = 33.33 ).

### Case 2 (Group RG / excellent)

**Fig. 5:**
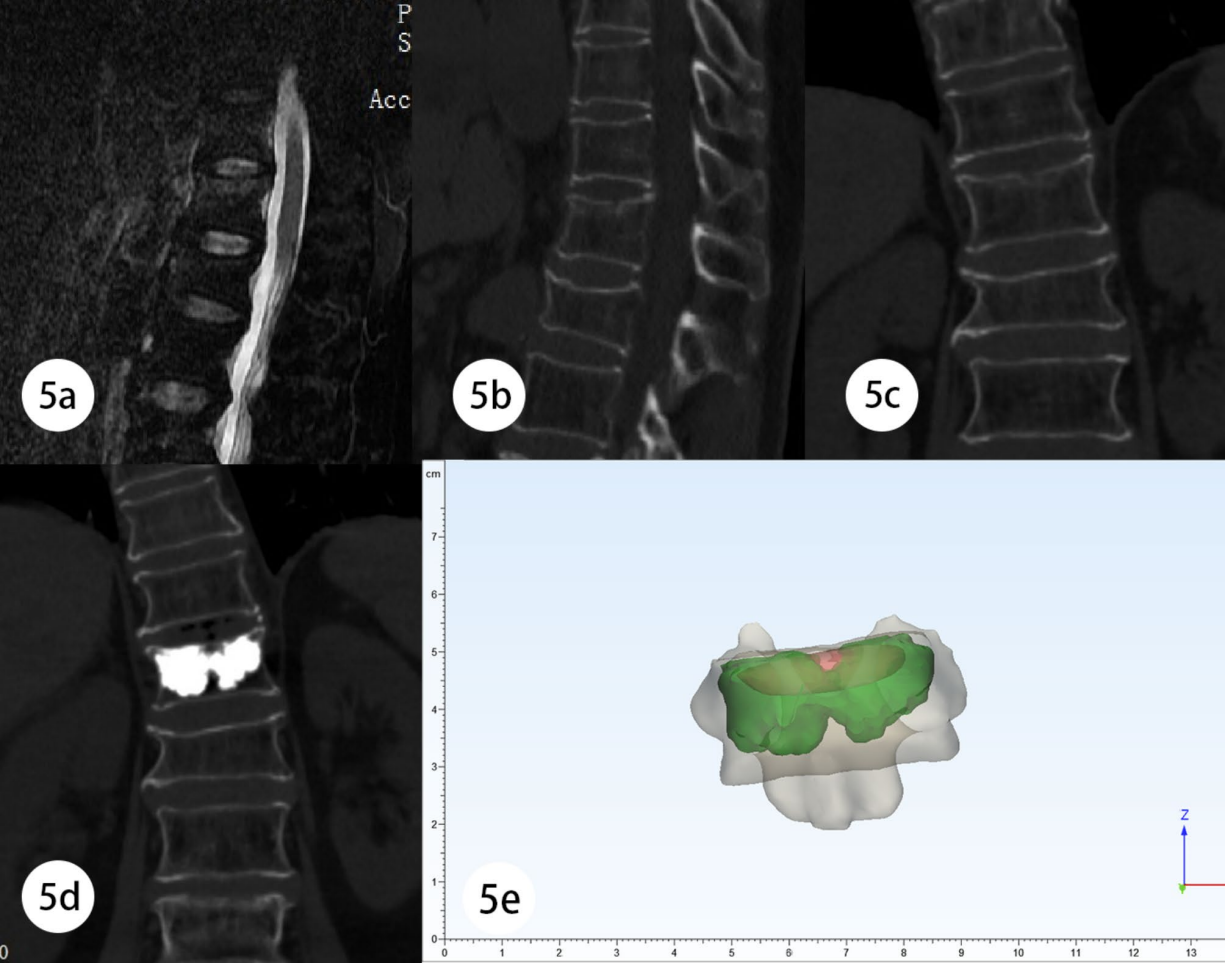
Case2: 5a The preoperative fat suppression image of thoracolumbar spine MRI showed SNs with Modic I changes in the cephalic endplate of T12 vertebra. 5b、c The sagittal and coronal images of preoperative CT. 5d Postoperative CT showed excellent distribution of bone cement. 5e Reverse reconstruction model, calculated R_f_=90.12%

A 71-year-old female complained of LBP for one week and denied any history of trauma or physical activity. Physical examination showed obvious T12 percussion pain and normal sensation and movement of bilateral lower limbs. MRI and CT confirmed the presence of SNs with Modic I changes on the cephalic endplate of T12 vertebra (Fig. 5a,b,c).

At admission, her NRS score was 6 and her ODI score was 71.11. After PVP, the patient felt significant pain relief (NRS = 2). According to the reconstruction model, we calculated her R_f_ value was as high as 90.12%, so she was classified into the ‘Excellent’ group (Fig. 5e,f). Until the last follow-up at 15 months after surgery, her NRS score was 2 and ODI score was 22.22.

## Discussion

The management of symptomatic SNs poses a challenge, and due to the limited efficacy of conservative treatment, many patients resort to intervertebral fusion as the ultimate solution for pain relief. Although spinal fusion surgery can yield satisfactory and enduring outcomes [17, 18], it may also result in loss of segmental motor function, greater trauma, and higher expenses. Therefore, surgeons must carefully weigh the risks and benefits of fusion surgery when dealing with elderly patients who are intolerant to general anesthesia and patients who require preservation of spinal motion.

The application of PVP surgery in this field has solved above problems very well. Previous researches have obtained satisfactory long-term effects on most patients [9–14]. We have described in detail the mechanism of PVP in treating symptomatic SNs in our previous studies [19]. Theoretically, this procedure enables rapid attainment of stability in the biomechanical microenvironment of SNs and inhibition of inflammatory cytokine release. In order to achieve the desired therapeutic effects, it is crucial that bone cement be distributed in appropriate locations. Consistent with previous research [6, 8], we recommend that bone cement should be used to fill the bone edema area surrounding SNs as fully as possible, creating a bowl-like enclosure [19].

 Based on our findings, it was observed that older symptomatic SNs patients were more likely to experience unfavorable clinical outcomes following PVP due to inadequate pain relief. The possible explanation is that older patients exhibit a higher degree of degeneration, which may interact with other factors to induce LBP, and PVP lacks therapeutic efficacy in this regard. In addition, we also found that patients with poor response tended to have lesions in the lower lumbar vertebrae. Theoretically, the physiological load increases gradually from the cephalic vertebra to the caudal vertebra, and accordingly, the strength and stiffness of the lumbar endplate are also gradually increased [20]. However, the existence of SNs greatly weakens the stress shielding effect of the endplate, and the vertical load can directly stimulate the pain receptors around the edematous bone marrow [7, 12, 19]. This pathological stimulation will not stop until Modic changes develop into type III to form sclerotic bone to block SNs. The therapeutic efficacy of PVP surgery lies in artificially accelerating the local sclerotic process, but inadequate cement distribution may compromise its intended blocking effect. Therefore, it is evident that patients with inadequate cement distribution exhibit significantly poorer clinical outcomes, particularly those with lesions situated in the lower lumbar vertebrae.

 To achieve a higher rate of bone edema filling, specific injection techniques are recommended by us, as well as other authors [9, 11–13]. Firstly, the trocar puncture direction should preferably be within the range of the bone edema area adjacent to SNs. Secondly, if there is no obvious leakage, the injection should be early to obtain better dispersion effect. Thirdly, if the initial distribution is undesirable, we frequently need to augment the dosage of bone cement in order to extend the dispersion range. In our study, although there was no statistical difference, the volume of cement in the Poor and n-RG groups was the smallest compared to the other groups, indicating that the dosage of cement is relatively insufficient in the less effective patients.

This study has several limitations that should be acknowledged. Firstly, its retrospective nature may increase the risk of inclusion bias. Secondly, due to the small sample size, we had to rely on current statistical methods; however, with a larger sample size, multiple regression analysis would likely provide more accurate results in exploring risk factors.

## Conclusion

The PVP procedure is a safe and effective treatment option for symptomatic SNs, providing rapid and lasting pain relief as well as functional improvement. However, advanced age and lower lumbar lesion location are associated with poorer outcomes. To achieve optimal therapeutic effects, it is crucial to fill the inflammatory edema area of the bone marrow with bone cement. Otherwise, it may bring more uncertainty to the clinical results.

## Data Availability

For any request for data and materials, please send a request to the corresponding author’s email address.

## Abbreviations

PVP: Percutaneous vertebroplasty
SNs: Schmorl’s nodes
R_f_: filling rate
RG: remission group
n-RG: non remission group
LBP: low back pain
MRI: magnetic resonance imaging
PKP: percutaneous kyphoplasty
BMD-T: bone mineral density T value
BMI: body mass index
PMMA: polymethyl methacrylate
CT: computed tomography
NRS: numerical rating scale
ODI: oswestry disability index
